# Intra-abdominal Mucormycosis in an Immunocompetent Host: A Rare Presentation and Literature Review

**DOI:** 10.7759/cureus.80730

**Published:** 2025-03-17

**Authors:** Sevag Hamamah, Nupur Savalia, Faizi Hai

**Affiliations:** 1 Internal Medicine, Scripps Mercy Hospital, San Diego, USA; 2 Gastroenterology, Scripps Mercy Hospital, San Diego, USA

**Keywords:** apophysomyces ossiformis, diagnostic challenges, gallbladder mucormycosis, liposomal amphotericin b, renal mucormycosis, septic shock

## Abstract

Mucormycosis is a severe, opportunistic infection caused by *Mucorales, *a taxonomical group of thermotolerant fungi primarily affecting the immunocompromised. Intra-abdominal involvement in mucormycosis is a rare entity, particularly in immunocompetent individuals. We present a fatal case of gallbladder and renal mucormycosis in an immunocompetent female, leading to septic shock and death. The diagnosis was confirmed via histopathology following cholecystectomy for suspected gangrenous cholecystitis and open right nephrectomy due to kidney infarction. Quantitative polymerase chain reaction of the tissue identified the presence of *Apophysomyces ossiformis.* The clinical picture was confounded by ongoing sepsis due to a *Klebsiella pneumoniae*-infected retroperitoneal hematoma*, *non-specific imaging findings, and the absence of traditional risk factors for mucormycosis, leading to a delayed diagnosis. Despite surgical debridement, initiation of liposomal amphotericin B with posaconazole, and aggressive treatment in the intensive care unit, the patient succumbed to complications of mucormycosis. Despite adequate antibiotic coverage, this case underscores the importance of considering Mucorales infection in otherwise immunocompetent patients with a deteriorating clinical condition. Early diagnosis and appropriate intervention are essential in enhancing mucormycosis survivability, though mortality rates remain high in severe cases.

## Introduction

Mucormycosis is a severe, opportunistic fungal infection caused by pathogens in the taxonomic group *Mucorales* including genera, *Mucor*, *Rhizopus*, *Rhizomucor*, *Apophysomyces*, *Cunninghamella*, *Saksenaea*, and *Lichtheimia* [1]. The primary risk factors are diabetes mellitus and immunocompromised status from causes including but not limited to hematologic malignancies, organ transplantation, neutropenia, iron overload, chronic immunosuppressant use, severe burns, or COVID-19 [2,3]. The condition has a high mortality rate (25%-87%), depending on the degree of underlying immunodeficiency and site of infection [4]. This is due to the angioinvasive ability of these fungi, leading to rapidly progressive tissue infarction and necrosis [4]. *Mucorales* are thermotolerant fungi, primarily found in soil and decaying matter, with routes of infection including fungal spore inhalation, ingestion, prior instrumentation, or direct skin inoculation [4]. Infection can involve multiple organ systems, notably rhinocerebral, pulmonary, cutaneous, gastrointestinal tract, and renal forms, listed in descending order of prevalence [5,6].

In immunocompetent individuals, these fungi are generally non-pathogenic due to their low virulence and the ability of host polymorphonuclear neutrophils (PMNs) to destroy the fungal pathogens via phagocytosis, oxidative metabolites, and fibrous networks [4,7]. Therefore, mucormycosis infection in immunocompetent individuals is rare, occurring at a rate of 4%-19% of total rhinocerebral infection cases [8]. Other forms of mucormycosis, such as pulmonary, cutaneous, and gastrointestinal, are even less prevalent in immunocompetent individuals, with exact incidence rates being poorly characterized in these populations.

More specifically, abdominal organ involvement is typically a manifestation of gastrointestinal or disseminated mucormycosis, primarily affecting the stomach (~57%) and the colon (~32%), with lesser involvement of the small intestine (~10%) and esophagus (~7%) [9]. Presentation is non-specific, with symptoms including abdominal pain, nausea, vomiting, and gastrointestinal bleeding. Renal involvement has been described in 20%-22% of disseminated cases, with case reports also describing the presence of isolated renal disease [6]. Liver, pancreatic, and gallbladder mucormycosis are extremely rare and underreported within the literature due to their overall low incidence.

Given its rarity, we present a fatal case of intra-abdominal mucormycosis that was found within the gallbladder and kidneys and diagnosed after laparoscopic cholecystectomy for suspected gangrenous cholecystitis in an immunocompetent female. Overall, this case highlights diagnostic challenges and the presence of competing diagnoses that contributed to a delay in diagnosis given the absence of traditional risk factors for mucormycosis.

## Case presentation

**Figure 1:**
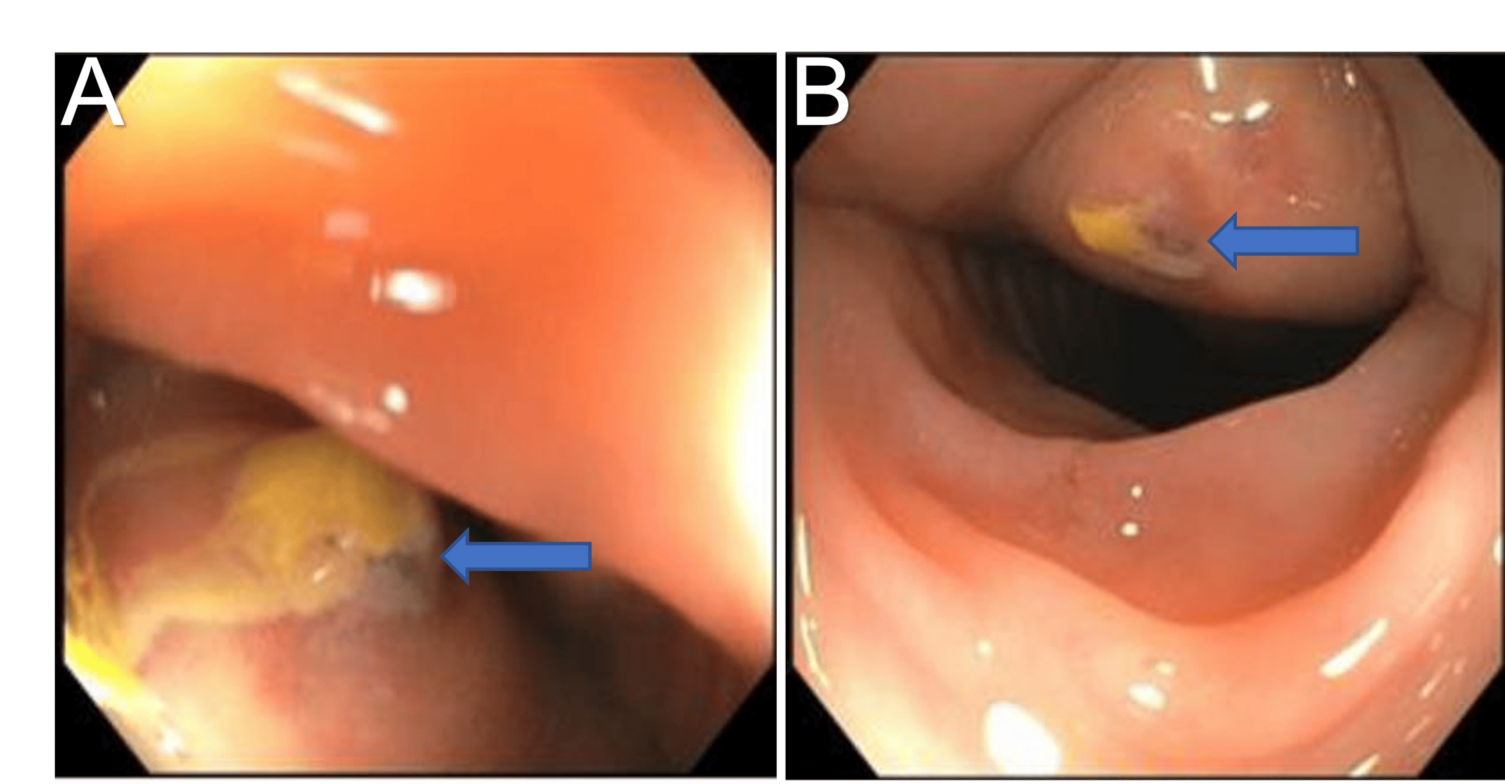
Endoscopic images of the lower gastrointestinal tract. Colonoscopy images are shown. (A) In the region of the splenic flexure, a 3-cm submucosal lesion was noted. (B) At around 5 cm distal to the first lesion, there was another 3 cm lesion with an umbilicated center. Arrows are included to highlight the submucosal lesions in the figures.

A 47-year-old immunocompetent female with history of Roux-en-Y Gastric Bypass (RYGB) performed 20 years prior to admission, anxiety, and well-controlled bipolar 1 disorder, was admitted with a month-long down-trend in hemoglobin from previous baseline of 12 g/dL to 8.5 g/dL. Her presenting symptoms included intermittent nausea, vomiting, and melena during this time frame, concerning for gastrointestinal bleeding. Patient did also endorse recent increase in Ibuprofen use. No other potential sources of overt bleeding such as menorrhagia or abnormal uterine bleeding were elicited on history taking. Esophagogastroduodenoscopy showed gastritis and an anastomotic ulcer while colonoscopy revealed two approximately 3 cm submucosal lesions with umbilicated center in the region of the splenic flexure (Figures 1A, 1B).

**Figure 2:**
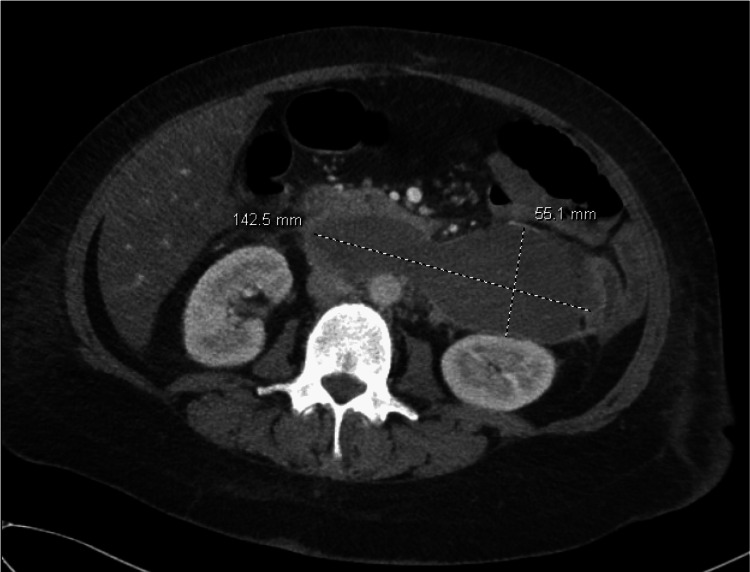
Computed tomography with and without contrast showing large retroperitoneal fluid collection. Anterior to the aorta and vena cava and posterior to the transverse duodenum there is a large bilobed fluid collection, which also abuts the descending colon. The fluid collection measures approximately 14.3 cm transversely and 5.5 cm anteroposteriorly. On imaging, the fluid collection appears to be denser than water. Image shown is contrast enhanced.

Subsequent computed tomography (CT) of the abdomen and pelvis was obtained after endoscopic studies did not find a convincing source of bleeding to explain the drop in hemoglobin. CT identified a large 14.3cm x 5.5cm retroperitoneal hematoma anterior to the aorta, posterior to the transverse duodenum, and abutting the descending colon (Figure 2). Further history did not reveal any specific causes that may explain the development of this retroperitoneal hematoma as the patient did not have any recent trauma, known vascular abnormalities or malignancy. 

**Figure 3:**
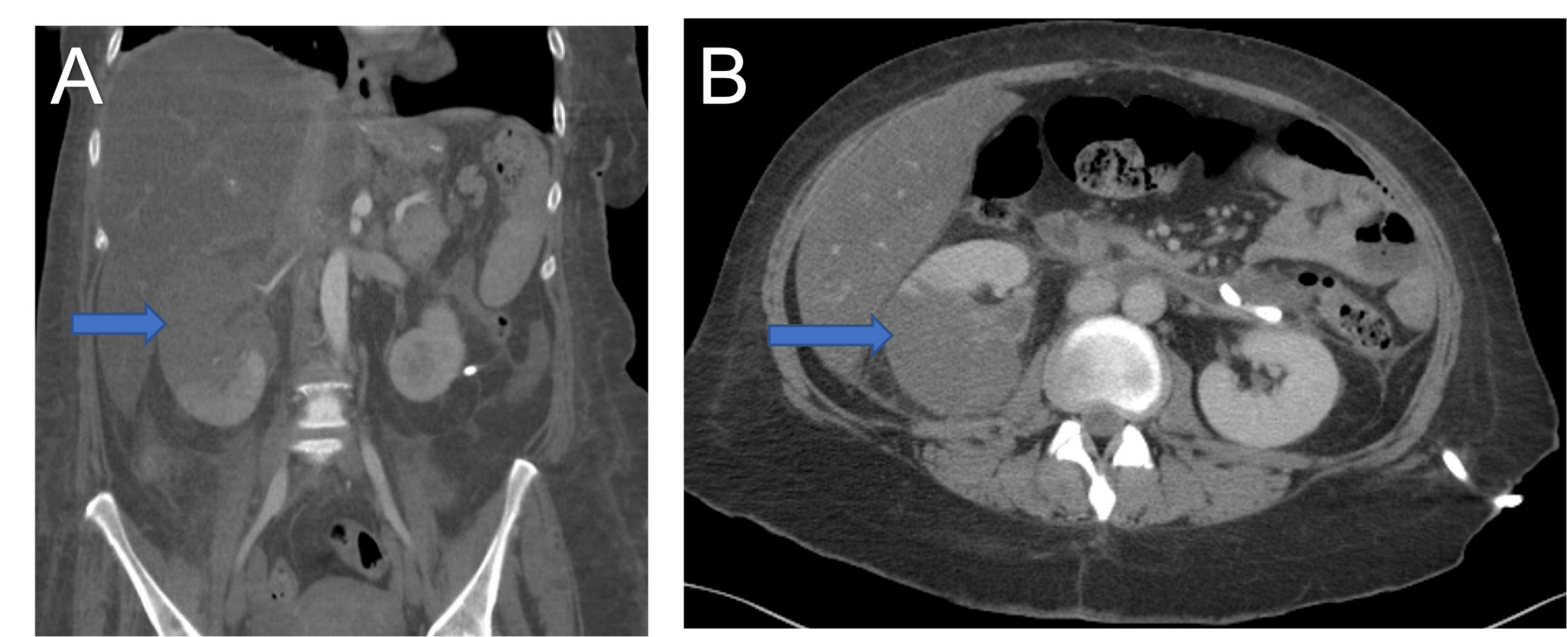
Computed tomography of the abdomen showing developing infarction of right kidney. The right kidney is enlarged with a large area of non-enhancement in the superior pole consistent with infarction. This scan is eight days following the computed tomography scan shown in Figure 2, which did not show any right kidney infarction. Retroperitoneal drain is present and can be seen exiting the site of the infected retroperitoneal hematoma, with evidence of fluid reduction and interval improvement. (A) Coronal and (B) axial views are shown to demonstrate the extent of infarction. Arrows in the figure point to the right kidney and area of infarction in both views.

Interventional Radiology (IR)-guided drainage of the retroperitoneal hematoma was performed, with intra-procedural cultures growing *Klebsiella* *pneumoniae*. Patient was started on ceftriaxone for pan-sensitive *K.* *pneumoniae*. Subsequent CT imaging eight days following initial drainage with drain placement showed interval improvement of the infected retroperitoneal hematoma along with a developing right renal infarct (Figures 3A, 3B). The patient then developed worsening leukocytosis (Table 1), tachycardia, and hypotension, which was initially responsive to fluid resuscitation. Over this period, hemoglobin continued to be stable around 7 to 8 g/dL without any evidence of overt blood loss. Despite adequate coverage with ceftriaxone and significant fluid reduction of the infected retroperitoneal hematoma, the patient continued to have persistent fevers and leukocytosis. Antibiotic coverage was escalated to include piperacillin-tazobactam; however, she soon progressed to septic shock. During work-up for other possible sources of shock, immunoglobulin E (IgE) level was significantly elevated to 1,908 IU/mL with worsening eosinophilia on manual differential (Table 1), which prompted empiric initiation of micafungin. 

**Table 1:** Leukocytosis trend with manual differential throughout hospital course. On admission, the patient had no leukocytosis. Patient started to develop leukocytosis around the time when CT imaging showed an infected retroperitoneal hematoma. Between hospital day 4 and 12, IR-guided drainage of the *Klebsiella pneumoniae* infected retroperitoneal fluid was performed and appropriate antibiotic was initiated. Despite adequate antibiotic coverage and significantly reduced size of the infected retroperitoneal fluid, there was worsening leukocytosis with eosinophilia that peaked on hospital day 30. At this time, mucormycosis was diagnosed and appropriate therapy, including liposomal amphotericin B and posaconazole, were initiated. Abbreviations: WBC, white blood cell count; %, percentage

Laboratory marker	On admission	Hospital day 4	Hospital day 12	Hospital day 24	Hospital day 30	Hospital day 35
WBC (cells/µL)	7.4	11.1	17.0	22.6	50.0	38.6
Neutrophil (%)	71.2	72.8	83.6	79.5	80.2	49.5
Lymphocytes (%)	19.9	15.3	7.5	6.6	4.2	10.1
Monocytes (%)	5.3	8.7	5.7	6.4	3.1	12.1
Eosinophils (%)	0.3	0.3	2.4	5.2	9.2	13.5

**Figure 4:**
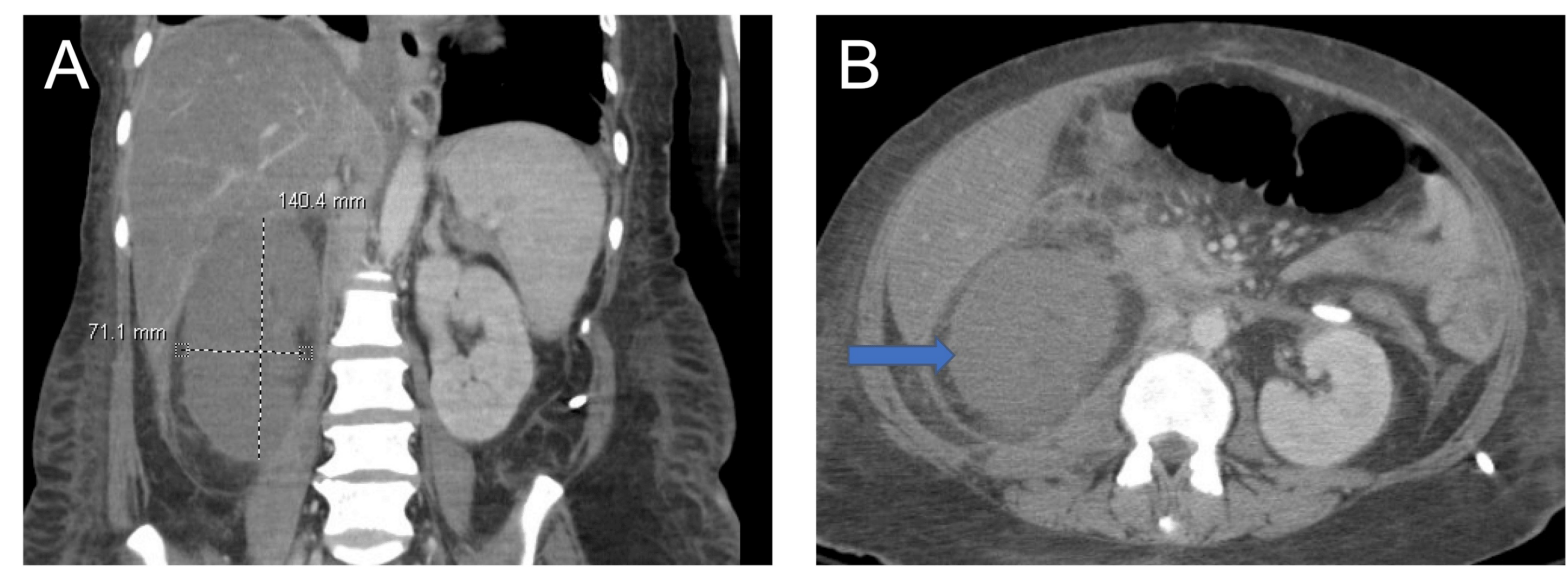
Computed tomography of the abdomen showing complete infarct of the right kidney. Abdominal imaging was repeated four days after the computed tomography scan in Figure 3. There is interval development of a complete right kidney infarction. (A) Coronal view demonstrating the complete right kidney infarction and the enlarged size of the right kidney (14 cm x 7 cm). (B) Axial view demonstrating the complete renal infarction. Arrow is included, pointing to the infarcted right kidney.

**Figure 5:**
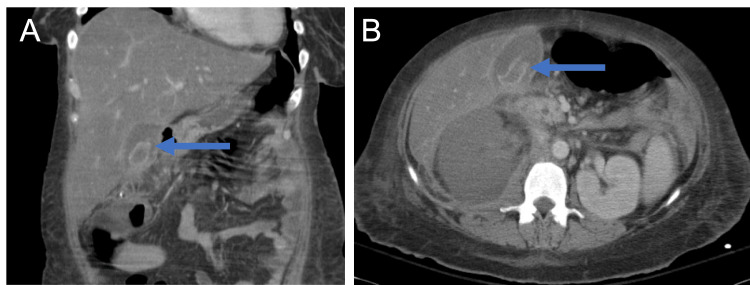
Computed tomography of the abdomen showing interval development of gangrenous cholecystitis. There is interval development of curvilinear membranes within the gallbladder concerning for gangrenous cholecystitis. (A) Coronal and (B) axial views are shown. Arrows are included in both views to highlight the gallbladder with curvilinear membrane development.

As part of the ongoing work-up, abdominal CT scans were repeated at 12 and 15 days following initial drainage and showed a now complete right renal infarct (Figures 4A, 4B), focal wall thickening of the right colon and development of gangrenous cholecystitis (Figures 5A, 5B). Given the patient's worsening condition, laparoscopic cholecystectomy was performed. Postoperatively, the patient remained in persistent shock requiring vasopressors with worsening metabolic and respiratory acidosis and minimal urine output requiring intensive care unit (ICU) level care. Emergent right nephrectomy due to the renal infarct was also performed two days after laparoscopic cholecystectomy.

**Figure 6:**
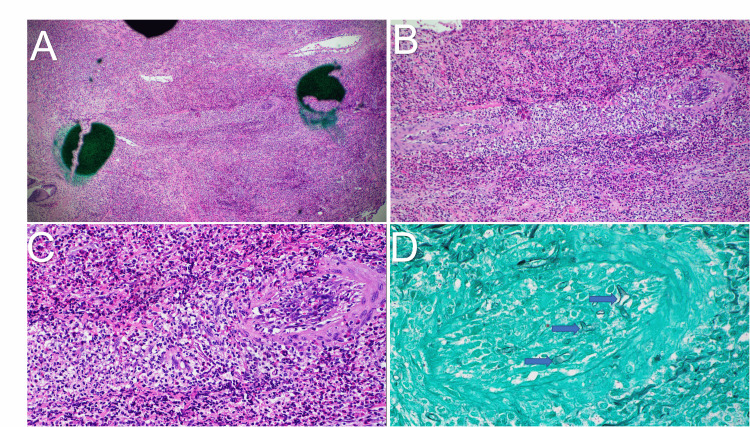
Gallbladder tissue histopathology images obtained following laparoscopic cholecystectomy. (A) A low power view of the gallbladder is shown. There is significant inflammation with abundant acute and chronic inflammation. The center of the image shows a small artery with necrotic changes. (B) A medium power view of the artery with necrotic changes is shown. (C) A high-power view with fungal elements, including broad and ribbon-like hyphae with inconspicuous septations and some branching is shown. (D) Grocott’s Methenamine Silver stain of the tissue is shown. Arrows are included in the figure to highlight the fungal elements.

Surgical pathology of the gallbladder revealed angioinvasive fungi with severe necrotizing inflammation, eosinophilia, and enzymatic fat necrosis, consistent with acute-on-chronic acalculous cholecystitis (Figures 6A-6D). Pathology also returned from the infarcted kidney showing necrosis with extensive inflammation and angioinvasive fungi with associated vascular thrombi. The fungal hyphae were morphologically suggestive of *Mucorales* species. Tissue samples were sent out to a tertiary center for PCR analysis, returning positive for *Apophysomyces* *ossiformis*.

Upon diagnosis of mucormycosis, antimicrobials were further broadened to include amphotericin B and posaconazole. Ongoing ICU management included volume resuscitation with packed red blood cells and fresh frozen plasma due to increasing bloody output from the abdominal Jackson-Pratt drain. A bicarbonate infusion was continued for metabolic acidosis, and a dialysis catheter was placed for emergent renal replacement therapy. Intubation with mechanical ventilation was performed due to worsening acidosis and shock. Stress-dose steroids were discontinued in the setting of disseminated mucormycosis, and broad-spectrum antimicrobials including anti-fungal medications were maintained. The patient's condition continued to deteriorate despite aggressive intervention and the patient ultimately succumbed to complications of mucormycosis 37 days into the hospitalization.

## Discussion

This unique case presentation describes an immunocompetent female who developed disseminated mucormycosis in the setting of an infected retroperitoneal hematoma with *K.* *pneumoniae*, leading to septic shock and death. Moreover, diagnosis of *Mucorales *infection was incidental and was revealed following laparoscopic cholecystectomy for suspected gangrenous cholecystitis, with surgical pathology showing angioinvasive fungi. To our knowledge, this is the first reported case of concomitant gallbladder and renal mucormycosis, confirmed through histopathological diagnosis.

The incidence of gallbladder involvement in mucormycosis is not well described in the literature, with only a few case reports documenting this phenomenon [10,11]. One case from 2011 describes an immunocompromised individual on chemotherapy presenting with suspected duodenal and biopsy-proven gallbladder *Mucor* involvement [10]. Duodenal ulcer perforation repair and cholecystectomy were attempted, though the patient passed away following septic shock and ICU management. There is another reported case of invasive intestinal mucormycosis in an immunocompetent host, with initial presentation of acalculous cholecystitis, though no histopathology results were reported, therefore it is unclear whether there was true gallbladder involvement [11]. In this case, however, the patient did have confirmed gastrointestinal involvement and did make a full recovery after ICU resuscitation and treatment with amphotericin B. Our presented case, in conjunction with the few reported cases within the literature, highlight variable presentations and outcomes about gallbladder involvement in mucormycosis.

Pathophysiology

As discussed, the incidence of gallbladder involvement in mucormycosis and direct mechanisms of gallbladder inoculation remain poorly characterized within the literature. Presumably, fungal spread to the gallbladder is a phenomenon resulting from disseminated infection, hematogenous spread, direct extension from adjacent infected tissue, nosocomial inoculation or sequelae of primary gastrointestinal mucormycosis. Abdominal organ involvement, via primary gastrointestinal inoculation, is thought to be secondary to ingestion of pathogens from fermented milk, dried bread products, fermented porridge, and corn-derived alcoholic drinks or via contaminated medical devices [12,13].

In our case, the initial source of involvement and route of inoculation is unclear. Although definitive diagnosis was confirmed via gallbladder histopathology, the chronology of renal infarction and extent of tissue necrosis of the right kidney when compared to the gallbladder, suggest that renal involvement preceded spread to the gallbladder. In this case, a plausible explanation for *Mucorales* fungi seeding into the abdomen is intra-procedural instrumentation during IR-guided drainage of the infected retroperitoneal hematoma, facilitating fungal inoculation into a favorable growth medium. This introduction into the abdomen can explain the chronology and rapid spread of the infection to other abdominal organs, such as the gallbladder and kidney. In general, the rate of nosocomial mucormycosis infections is not well characterized. One tertiary care center study in India described a 9% rate of mucormycosis infections that were determined to be a result of instrumentation or contaminated medical devices [14]. Further, the largest literature review of 169 healthcare-associated mucormycosis cases describes nosocomial infections primarily manifesting as cutaneous mucormycosis (57%) with the second most common localization being the gastrointestinal (15%) [15]. The portal of entry and outbreaks in this study were attributed to medical devices or adhesive tapes, with specific mention of adhesive bandages, wooden tongue depressors, ostomy bags and water circuitry damage as potential culprits [15]. There is also a case series of five patients that underwent various surgical interventions with postoperative courses complicated by severe abdominal wall mucormycosis, mimicking necrotizing fasciitis, though no involvement within the abdominal cavity was described [16]. Therefore, surgical and procedural tools may also facilitate inoculation of *Mucorales *infection.

Further, the rapidly progressive right renal infarction seen on imaging and widespread renal necrosis on pathology was almost certainly a result of angioinvasion of fungal hyphae. Similarly, gallbladder histopathology also demonstrated evidence of angioinvasion. This is evident through extensive inflammation and necrotic changes within the central artery with associated fungal elements shown in the high-power view in Figure 6. Though uncommon, renal involvement in mucormycosis is better described within the literature than that of the gallbladder [17-22], with a majority of reported cases stemming from India. In a case series of 15 isolated renal mucormycosis cases in immunocompetent hosts, CT images exhibited similarities to our findings of the right kidney, with 100% of those cases showing enlarged globular kidneys with patchy, decreased or absent enhancement resulting from angioinvasion, vascular thrombi formation and infarction [6]. Interestingly, 46.1% of cases demonstrated associated renal abscess and 30.7% with perinephric fluid collection [6]. Though this was not seen in the right kidney, there was a perinephric fluid collection in the left kidney in our case. However, cultures were not specifically sent for fungus at the time of drainage. Further, bilateral renal involvement was seen in a smaller subset of the patients (4/15) in the case series [6]. Interestingly, all 15 of these patients were on IV antibiotics with primary diagnosis of bacterial sepsis due to pyelonephritis with unrelenting fever and persistent leukocytosis. The patient in our case presentation instead had an infected retroperitoneal hematoma and related perinephric abscess that confounded the clinical picture.

Overall, multiple pathogenic explanations are proposed in the literature that support the rapid intra-abdominal spread, manifesting as renal and gallbladder mucormycosis as a sequela of seeding via instrumentation in this case presentation. These mechanisms include the angioinvasive properties of *Mucorales *fungi, related tissue destruction and concurrent host immune dysfunction [7]. Following germination, *Mucorales* fungal hyphae have a propensity to invade endothelial cells and penetrate blood vessel walls via interaction with endothelial receptors [23]. The consequence of angioinvasion is multi-fold, including an increased risk of hematogenous dissemination, local endothelial apoptosis and vascular inflammation [7]. These processes activate clotting cascades, contribute to tissue ischemia, and promote blood vessel occlusion. Resulting tissue infarction and subsequent necrosis via hydrolytic enzyme production mediated by these fungi further creates a hypoxic and acidic microenvironment that enhances *Mucorales* survivability [24]. Immunocompromised status impairs host defense, with inability of PMNs and macrophages to clear fungal spores through oxidative and non-oxidative mechanisms [25].

Although no traditional risk factors related to immunosuppression were present in this case, initial *Klebsiella* infection, broad-spectrum antibiotic use and related sepsis may have increased invasive fungal overgrowth [26,27]. It is also important to note that our patient has a history of RYGB with inconsistent use of vitamins which may indirectly cause functional immunosuppression and cytopenia within hematologic cell lines, primarily through nutrient malabsorption [28]. Supplementation of appropriate vitamins, even in sub-optimal doses, is found to prevent the probability of vitamin deficiency up to at least 12 years following RYGB [29]. Specific nutrients deficiencies that are implicated post-gastric bypass include iron, zinc, folate, along with vitamins B1, B12, A, K, D, and E [28]. Workup for nutrient malabsorption in our case revealed elevated vitamin B12 with normal serum homocysteine and methylmalonic acid levels. Vitamin D levels were low and folate was within normal limits while other listed vitamins and minerals were not checked. Of these vitamins, B12 and Vitamin D are found to have associations with immunomodulatory processes [30,31]. For example, Vitamin D receptors are expressed on adaptive immune cells, with the ability to affect innate and adaptive immune responses [32]. Similarly, vitamin B12 supplementation in deficient individuals is shown to augment immune system regulation via enhancing T lymphocytes and natural killer cell responses [31]. Though vitamin deficiency does not directly constitute immunocompromised status, this is an important consideration in this case which may have played a role in increasing susceptibility to severe infection. Additionally, the interrelationship between bariatric surgery and its influence on gut microbial composition has been elucidated [33], though immunomodulatory roles in the long-term are not well studied. As such, it is difficult to determine the extent to which the patient’s history of RYGB contributed to this case presentation.

Diagnostic challenges

Multiple diagnostic and therapeutic challenges precluded early diagnosis of mucormycosis in this case. The patient’s initial presentation of suspected gastrointestinal (GI) bleeding, particularly in an immunocompetent host, is an atypical presentation to suspect mucormycosis. In a meta-analysis of 851 patients with mucormycosis, GI involvement was reported in 7% of total cases, with individuals with previous solid organ transplantation being at the highest risk of developing GI mucormycosis [5]. Similarly, another meta-analysis of 929 mucormycosis cases reported a similar rate of GI mucormycosis at under 10% of total cases [34]. These meta-analyses include mostly immunocompromised individuals, therefore, suspicion for gastrointestinal mucormycosis in an immunocompetent individual is generally even lower.

Upon ruling out gastrointestinal bleeding, CT imaging revealed a retroperitoneal hematoma with adjacent perinephric abscess. Drainage and culture of the sample confirmed the presence of *K.* *pneumoniae*. Given overlapping features with bacterial sepsis and a clear source, appropriate antibiotics were initiated, leading to a delay in consideration of fungal pathogens as a primary contributor of ongoing sepsis. Antibiotics were broadened throughout the admission as fevers and leukocytosis did not resolve. During this time, CT imaging searching for other intra-abdominal sources of sepsis, showed gangrenous cholecystitis and right renal infarct, which were interpreted as complications of critical illness rather than fungal dissemination. This misperception was, in part, due to the rarity of mucormycosis affecting the gallbladder and kidney, particularly in immunocompetent hosts.

Clues to fungal involvement in this case included a markedly elevated IgE level, worsening eosinophilia and persistent fevers with leukocytosis despite broad-spectrum antibiotic coverage. Although micafungin was initiated to cover for concurrent fungemia, its efficacy against mucormycosis is controversial, as *Mucorales* are intrinsically resistant to echinocandins through their inherent serine/threonine phosphatase activity [35]. Further, detection of *Mucorales* fungi in blood cultures is often difficult as these fungal pathogens grow slowly in cultures even in disseminated disease [36]. Therefore, blood cultures are of limited utility in diagnosis of *Mucorales* fungemia [37] and did not grow any fungi throughout the hospitalization.

Current diagnostic options for mucormycosis include direct microscopy, bronchoalveolar lavage, histopathology, fungal cultures, molecular and serologic testing [38]. The use of certain diagnostic tools, depending on the location of involvement in the body [38]. Definitive diagnosis is made through histopathology showing broad, aseptate hyphae with right angle branching, however visualization of these hyphae may be obscured by dense inflammation, sheets of acute inflammatory cells, vascular hemorrhage, and tissue necrosis [39]. Periodic acid-Schiff (PAS) or Grocott-Gomori methenamine silver (GMS) staining is used on histopathology slides to enhance visibility of *Mucorales* fungi [40]. In combination with histopathology, utilizing fungal cultures and direct microscopy are instrumental in confirming diagnosis [41]. Fungal growth occurs on Sabouraud dextrose agar, typically taking three to seven days for a positive result. However, it should be noted that in one study of 587 patients that had biopsy proven mucormycosis, 79% exhibited fungal culture positivity, while the rest yielded false negative results [5].

Further, studies have assessed the utility of serologic and molecular testing in the form of quantitative polymerase chain reaction (qPCR) with improving efficacy over the years [42]. Prospective study results of 232 patients with mucormycosis showed qPCR testing with sensitivity of 85.2% and specificity of 89.8% [43]. Sensitivity of qPCR testing continues to be refined with 2025 findings showing that using larger sample volumes for nucleic acid extraction and DNA template volumes increases sensitivity to around 95.5% [44]. Importantly, mucormycosis qPCR is not widely standardized or readily available in all hospital laboratories including the hospital where this case took place, often requiring specialized laboratory and expertise in molecular diagnostics [38].

Serum β D-glucan and galactomannan assays are less useful in identifying mucormycosis due to a lack of β D-glucan and galactomannan polysaccharide in *Mucorales* cell walls, though can help exclude presence of other invasive fungi like aspergillosis and candidiasis [45]. Lastly, gastrointestinal tract mucormycosis can present with ulcerations, necrosis or black eschars in affected segments seen by direct visualization via endoscopy [46]. There were submucosal lesions seen in colonoscopy early in the clinical course, with development of ascending colon colitis as severity of illness progressed, though these were never confirmed to be related to mucormycosis.

Given the complex presentation, definitive histopathologic diagnosis was delayed and occurred incidentally postoperatively following cholecystectomy with surgical pathology showing fungal hyphae with vascular thrombi. These findings were redemonstrated on histopathology of the infarcted kidney. Due to low suspicion of mucormycosis, comprehensive fungal testing was not done early in the hospitalization. Fungal qPCR was eventually completed, requiring sample transfer to a tertiary laboratory, with *A.* *ossiformis* ultimately being detected via qPCR from gallbladder and kidney tissue samples. Pleural fluid was also sent out with negative results for fungi. Ultimately, this diagnosis was made nearly 30 days following admission due to complicating factors in the clinic picture, contributing to mortality at day 37 of hospitalization in this patient.

Management and outcomes

The mainstay of treatment of mucormycosis includes early surgical debridement, liposomal amphotericin B and addressing underlying risk factors [47,48]. Upon diagnosis, appropriate therapy was initiated for this patient including liposomal amphotericin B 500 mg and posaconazole 300 mg twice daily followed by 300 mg daily for disseminated mucormycosis. In general, first-line therapy with liposomal amphotericin B, dosed at 5-10 mg/kg/day intravenously with consideration of higher doses for more severe mucormycosis infection [49]. Dosages of higher than 10 mg/kg/day IV are shown to increase risks of nephrotoxicity, without increasing serum concentration of the drug. Further, combination therapy with posaconazole or echinocandins has been suggested, though the evidence is inconclusive [50]. Of the combination therapies, amphotericin B and posaconazole have shown to be the most efficacious in treatment success, however, the evidence is weak and extrapolated from case reports with studies primarily assessed in rhinocerebral mucormycosis [51,52]. Further, efficacy of adding a combination agent likely depends on the severity of the disease, involved anatomy, as well as specific *Mucorales* spp*.* that is being targeted [52]. Therefore, posaconazole is indicated as either a step-down therapy from amphotericin B or as a salvage therapy in conjunction with amphotericin B in severe, late-stage cases such as the one presented [48].

Due to the angioinvasive nature of *Mucorales* fungi and resulting ischemic tissue necrosis, both drug delivery and leukocyte migration to affected tissues can be compromised, worsening outcomes [48]. Therefore, in conjunction with antifungal therapy, early and aggressive surgical debridement should be highly considered to debulk infection in involved organs [53]. In a meta-analysis of 929 mucormycosis cases, overall survival was found to be 61% in those treated with anti-fungal therapy alone, 57% in individuals treated with surgery alone and 70% for those with combination therapy with both anti-fungal therapy and surgical debridement [34]. In gastrointestinal mucormycosis specifically, analysis of 19 cases indicated a survival rate of around 50% in those treated with amphotericin and surgical debridement [54]. In another systematic analysis, cases with renal involvement (n = 10) exhibited a 100% mortality rate in those that did not undergo surgery, compared to 38% that did [55]. Interestingly, mortality rates of individuals with disseminated mucormycosis in the same analysis (n = 24), were similar despite whether they underwent surgical intervention or not [55]. No data exists regarding survival rates following combination treatment in individuals with gallbladder involvement specifically. Despite combination therapy with surgical debridement, amphotericin B and posaconazole, this patient unfortunately passed away due to severe mucormycosis infection, with delayed diagnosis and initiation of therapy being a contributing factor.

*Apophysomyces* *ossiformis*

*Apophysomyces* are a rare, but highly virulent genera that cause mucormycosis, with a higher propensity to cause severe disease in immunocompetent hosts than most other *Mucorales* fungi, besides *Saksenaea* [56]. This genus has been primarily known to exert its pathogenicity after direct cutaneous inoculation, leading to necrotizing soft tissue infections, with potential for hematogenous dissemination [57]. To date, a majority of cases are caused by trauma-related inoculation (~55%) with contact of contaminated material. Healthcare-associated mucormycosis cases caused by *Apophysomyces* contribute to a smaller subset of infections [56,57]. Interestingly, case reports from the 1990s and early 2000s describe renal involvement from *Apophysomyces *spp*. *following trauma and cutaneous inoculation [58,59]. Additionally, there is one case reported of *A. elegans* infection from an unknown source of entry in an immunocompetent male causing renal mucormycosis and eventual disseminated disease contributing to patient’s death [60]. In the obtained history from our case, there was no recent evidence of trauma and no sites of cutaneous involvement seen on physical exam. Further, there are no previously reported cases of intra-abdominal *A. ossiformis *stemming from nosocomial inoculation. However, there is a reported case of severe gangrenous cutaneous *A. elegans* infection following instrumentation during abdominoplasty and liposuction, without any extension to the abdominal cavity [61]. Therefore, the isolation of *A. ossiformis *in our case with intra-abdominal involvement serves as another unique element of this case presentation of mucormycosis.

## Conclusions

To summarize, gallbladder and renal involvement in mucormycosis is a rare entity, particularly in a host without known immunocompromising factors. Our case underscores an atypical, but fatal presentation of mucormycosis infection, confounded by ongoing bacterial sepsis, non-specific imaging findings and incidental histopathological diagnosis of *A.* *ossiformis* infection, confirmed by qPCR testing. Despite its rarity, we highlight the need for heightened suspicion of invasive fungal infections in critically ill patients that continue to deteriorate despite otherwise appropriate antibiotic therapy, even in those that are otherwise immunocompetent. Further, nosocomial acquired *Mucorales* via contaminated medical equipment should be considered in individuals with prolonged hospitalization and recent instrumentation. Vascular complications such as infarctions and gangrene may also be sequelae of invasive fungal infection and could be considered in the right clinical context. Though histopathology and culture are the gold standard for diagnosis, PCR testing continues to improve and can be used to guide diagnostic acumen. Early surgical debridement and liposomal amphotericin B remain the mainstay of treatment. Overall, timely intervention is critical when treating mucormycosis and increasing awareness to expedite diagnosis and treatment can improve outcomes, though mortality rates remain high.
